# Targeted nanobody complex enhanced photodynamic therapy for lung cancer by overcoming tumor microenvironment

**DOI:** 10.1186/s12935-020-01613-0

**Published:** 2020-11-27

**Authors:** Qing Zhang, Lian Wu, Shaozheng Liu, Qingjie Chen, Lingpeng Zeng, Xuezhong Chen, Qing Zhang

**Affiliations:** 1 Department of Nuclear Medicine, The First Affiliated Hospital of Nanchang University, No. 17, Yongwai Zheng Street, Donghu District, Nanchang, 330006 People’s Republic of China; 2 Department of Nephrology, The First Affiliated Hospital of Nanchang University, Nanchang, 330006 People’s Republic of China

**Keywords:** IR1048MZ, Anti-EGFR-nanobody, NIR-II imaging, Hypoxic enhancement, Photodynamic therapy

## Abstract

**Background:**

To investigate the efficacy of a PLGA-based nanobody complex in photodynamic therapy (PDT) and NIR-II imaging in A549 tumor hypoxic model.

**Method:**

IR1048-MZ was firstly synthesized by conjugating a nitro imidazole group to IR1048. IR1048-MZ and Cat were then encapsulated in PLGA-SH solution. Anti-EGFR-Nanobody was also expressed and purified, and finally Anti-EGFR-Nanobody@PLGA-IR1048MZ-Cat (Nb@IC-NPs) nanobody complex was obtained based on the formation of desulfide bond between PLGA-SH and Anti-EGFR-Nanobody. Size distribution and morphology were characterized by TEM and DLS. Spectrum of Nb@IC-NPs towards NTR was measured by UV and fluorescence, while the particle’s selective response was studied using fluorescence. The uptake of Nb@IC-NPs in A549 cells was observed by flow cytometry and CLSM. In the meantime, its’ catalytic ability that decomposes H_2_O_2_ both extra-and intra-cellular was observed by fluorescence and CLSM. In vitro photodynamic toxicity of Nb@IC-NPs was examined by MTT, Live/Dead Cell Staining, Flow Cytometry and Apoptosis Assay. Tumor-bearing model was constructed to observe a semi-quantitative fluorescent distribution and the possibility of NIR-II fluorescence/photoacoustic (PA) imaging. Effect of Nb@IC-NPs on enhancing A549 tumor hypoxia and expression profile of HIF-1α was investigated in the presence of NIR. An A549 tumor metastasis model was also constructed to confirm the complex’ potential to destroy primary tumor, inhibit lung metastasis, and prolong mice’ survival. Lastly, impact of Nb@IC-NPs on mice’ main organs and blood indices was observed.

**Results:**

Nb@IC-NPs was successfully fabricated with good homogeneity. The fluorescent absorbance of Nb@IC-NPs showed a linear relationship with the concentration of NTR, and a higher concentration of NTR corresponded to a stronger photoacoustic signal. In addition, Nb@IC-NPs showed a stable selectivity toward NTR. Our results also suggested a high efficient uptake of Nb@IC-NPs in A549 cells, which was more efficient than IC-NPs and IR1048-MZ alone. In vitro assays confirmed the effects of Nb@IC-NPs on catalytic O_2_ generation even in hypoxic cells. The cell viability was upregulated with the nanocomplex at the absence of the laser, whereas it was dramatically declined with laser treatment that excited at 980 nm. Nb@IC-NPs achieved tumor hypoxia NIR-II/PA imaging through assisting A549 gathering. When NIR was applied, Nb@IC-NPs can significantly relieve A549 cellular/tumor hypoxia by generating more reactive oxygen species (ROS), which in turn helps lower the expression level of HIF-1α. In summary, Nb@IC-NPs based PDT can efficiently decimate A549 primary tumor, inhibit metastatic lung cancer, and prolong the lifespan of the mice under tolerable dosage. At last, in vivo toxicity tests of the nanocomplex showed its biosafety to the main organs and normal blood indices values.

**Conclusion:**

Nb@IC-NPs improves tumor hypoxia through catalytic reaction and lowers the expression level of HIF-1α. It achieves tumor PA imaging through intensified NIR-II fluorescence signal that caused by response of the complex to the lesion’s nitroreductase (NTR). Nb@IC-NPs based PDT can efficiently kill A549 primary tumor, inhibit a lung metastasis, as well as prolong mice’ survival cycle.

## Background

Lung cancer is the most commonly diagnosed cancer and the leading cause of cancer mortality worldwide, and non-small cell lung cancer (NSCLC) is the most common subtype which accounts for about 85% of lung cancers [1, 2]. Removal of malignant tumors through surgery has been considered as the best treatment option for NSCLC, which promoted long-term response and survival for about 30% of the patients [3, 4]. Most of NSCLC patients have responded effectively to chemo-, radiation and adjuvant-therapies in clinic [5–7]. However, these traditional treatments are limited by development of recurrence, chemoresistance, and non-specific targeting, resulting in survival time reducing [8, 9]. Promisingly, safer and more effective therapeutic modalities have been widely developed through nanoparticle (NP)-mediated photodynamic therapy (PDT) [10, 11]. Phototherapy is kind of less invasive treatment induced by light, preferably near-infrared (NIR) light with superior tissue penetration ability [11]. Usually, it may involve phototherapeutic agents that are able to selectively kill cancer cells via generation of singlet oxygen under light irradiation, without high toxicity or much damage to normal tissues in dark [12, 13]. Photosensitizer-carrying nanoparticles could increase the water solubility of photosensitizer molecules, enhance their tumor accumulation, thus improving the therapeutic efficacy and specificity of PDT [14, 15]. Over the past decade, nanoparticle-based PDT have shown great promise in treatment of cancer, including lung cancer [16–18], achieving encouraging therapeutic efficacies [19, 20]. In addition, nanotechnology provides a platform for the integration of multiple functionalities in a single construct with effective targeting, achieving the purpose of tumor imaging and diagnosis and treatment [21, 22]

However, the effectiveness of PDT is impaired by hypoxia, which was recognized as one of the hostile characteristics of solid tumors [23, 24]. Further oxygen consumption in the proceeding of PDT will result in aggravation of tumor hypoxia, leading a low therapeutic efficiency [25]. Much effort has been devoted to promote the intracellular generation of oxygen by decomposing H_2_O_2_, which is thought to be an effective strategy [26–28]. Beside simply relying on the enhanced permeability and retention (EPR) effect for tumor passive tumor, the well-engineered phototherapeutic nanoagents were conjugated with targeting ligands, including anti-epidermal growth factor receptor (EGFR) antibody, to selectively target tumor via active tumor homing [29]. Recently, nanobodies (Nbs) have shown great advantages when compared with traditional antibodies in targeted drug delivery, and demonstrated high potential in clinical application [30]. Nb, a distinct type of antibody fragment, is derived from heavy-chain-only antibodies that circulate in sera of camelids. Nbs are more amenable to faster engineering and cheaper production, and exhibit enhanced tissue penetration and lower immunogenicity [31]. The superior stability, high affinity, high target specificity, and fast clearance of Nbs offer special advantages compared to conventional antibodies in application of noninvasive diagnostic imaging and tumor therapy [31–33]. Nb is able to mediate the endocytosis of specific receptors, such as EGFR, in tumor cells, promoting Nb as tumor-targeting ligands for drug nanocarrier designed in targeted cancer therapy and diagnosis [34–36]. In addition, anti-EGFR Nb also shows tumor suppressing properties, which further enhance the efficacy of drug delivery [37, 38]. Nevertheless, short blood half-life due to its small size limits its therapeutic properties.

Herein, a multi-functional nanoplatform Nb@IC was designed by using of anti-EGFR Nb combined with photosensitizer and catalase (Cat) to improve tumor hypoxia, enhancing the therapeutic efficacy of PDT of IR1048MZ in lung cancer. IR1048-MZ was synthesized by conjugation with a nitro imidazole group as a specific hypoxia trigger with IR-1048, a dye as NIR-II/photoacoustic (PA) signal reporter [39]. IR1048MZ and Cat were encapsulated into sulfhydryl modified PLGA polymer (PLGA-SH), which conjugated with purified anti-EGFR Nb by disulfide linkage, to obtain EGFR-Nanobody@PLGA-IR1048MZ-Cat nanoparticles (Nb@IC-NPs). Nb@IC-NPs showed good biocompatibility, which could be efficiently uptaken by A549 cells, achieving sufficient oxygen supply and the reduction of Hypoxia-inducible factor-1α (HIF-1α) expression in A549 tumor. Moreover, Nb@IC-NPs was sensitive to nitroreductase (NTR) enzyme in the tumor tissues, resulting in enhanced NIR-II fluorescence signal, which enables tumor diagnosis and real-time tumor hypoxia imaging. In the current study, we sought to characterize the effects of Nb@IC-NPs-based PDT on the growth of subcutaneous A549 tumors, lung metastasis, therefore providing a new approach to the treatment of NSCLC.

## Methods and materials

### Materials

mPEG-SS-PLGA-SH was purchased from Ruixi Biological Technology (Xi’an, China); Catalase (CAT) solution (≥ 35, 000 units/mg protein) was purchased from Aladdin (China); IR1048, PEG-NH2 (5 K), and 5, 5′-Dithio bis-(2-nitrobenzoic acid) (DTNB) were purchased from Sigma-Aldrich (St Louis, MO, USA); DMF, 2-Nitroimidazole, Dichloromethane, and NADH (Nicotinamide adenine dinucleotide) were purchased from Johnson & Johnson (Beijing, China); Transglutaminase (mTGase) was provided by Yiming Biological (Jiangsu, China); Calcein-AM/Propidium Iodide (PI) and Hoechst 33342 was obtained from Yeasen Biotechnology (Shanghai, China); Penicillin–Streptomycin mixture, Fetal Bovine Serum (FBS), dulbecco's modified eagle medium (DMEM) and Trypsin-Ethylenediaminetetraacetic acid (EDTA) were purchased from Life Technologies (Carlsbad, USA); Dialysis bag was manufactured by Beyotime Biotechnology (Shanghai, China); MTT kit was purchased from Solarbio Technology (Beijing, China); A549 cell line containing Luciferase was purchased from American Type Culture Collection (ATCC; Manassas, VA, USA); all other reagents included in this study were analytical grade.

### Methods

#### Expression and purification of nanobody

Anti-EGFR-Nanobody was constructed based on recent report [40]. Plasmid Nb-C3-Q was constructed, including Anti-EGFR-Nanobody 7D12 fragments with His6 tag on N-terminal and GGGGS-CCC-GSGSGS-LLQS on C-terminal, while -LLQS tag was designed to recognize mTGase and -CCC- as a chemical modification for the protein. Plasmid was transformed into the Rosetta-gami cell in E.Coli, and then induced to be expressed by IPTG by an improved method reported recently [41]. Cells were collected by centrifugation at 4000 rpm and resuspended with buffer (20 mM Tris–HCl, 150 mM NaCl, pH 8.0), followed by ultrasonic processing on ice. Cell lysate was centrifuged at 16,000 rpm for 30 min at 4℃. The recombinant protein 7D12-C3-Q was purified using a Ni–NTA Chromatographic column (His-Trap HP column 5 mL, GE Healthcare) (Linear gradient: 40–250 mM Imidazole, 20 mM Tris–HCl, 150 mM NaCl, pH 8.0). PEGylation was initiated by adding mTGase (0.5–1 U/mL) at room temperature. Nb-C3-Q (3 mg/mL) and PEG-NH_2_ (1 mg/mL) were latter incubated in PBS containing 1 mM Ascorbic acid so as to finish the rest steps. After 1 h incubation, products were analyzed by SDS-PAGE (15%). The PEGylated nanobody [Nb(PEG)] was purified by gel filtration, using ӒKTA purification system that’s mainly rely on a HiLoad 10/60 Superdex 200column.

#### Synthesis of IR1048MZ

NIR-II fluorescence dye IR1048-MZ was synthesized as recently performed [39]. While being stirred, sodium hydride (0.65 g, 16.3 mmol) was added portion-wise into DMF (20 mL) containing 2-aminoimidazole sulfate (1 g, 3.8 mmol), during which ice bath was used to keep temperature under 30℃. After being stirred for 30 min at 25–30 ℃, the whole solution was cooled down to 0℃. Tert-Butyl-N-(2-bromoethyl) carbamat (1.7 g, 7.6 mmol) was dissolved into dichloromethane with DMF (1 mL) added. Ice bath was then removed after being stirred for 1 min, then the solution was left being stirred for 16 h at room temperature. Reaction was quenched gently by water (10 mL) and then extracted by dichloromethane (EtOAc, 4 × 10 mL). The product was filtered after dried by sodium sulfate and filtrate was concentrated till only residue left. Residue was preserved < 0.5 mm Hg for 24 h in order to remove leftover DMF, after which normal-phase chromatography was performed (Methanol/water = 100:0 (0 min) to 20:80 (20 min); flow rates = 1 mL/min) to obtain MZH-BOC. TFA (2 mL) was added into MZH-BOC (11.3 mg, 0.05 mmol), containing dichloromethane (2 mL) at 30℃. Solvent was evaporated under reduced pressure till concentrated residue showed up. Final product can be of direct use without further purification after preserved 24 h at 30 °C in a vacuum oven. IR-1048 (37 mg, 0.05 mmol) was dissolved in anhydrous DMF (10 mL), after which MZH (63.1 mg, 0.5 mmol) was added drop-wise under vigorous stirring. The whole mixture was then stirred for 4 h at 30℃ in the protection of N_2_. Crude product was obtained after solvent was evaporated under reduced pressure. Lastly, crude product was purified by dichloromethane and methanol using silica gel column chromatography until IR1048-MZ was obtained.

#### Preparation of Nb@IC-NPs

Briefly, IR1048-MZ (4 mg) and Cat (4 mg) were dissolved in DMSO (200 mg), and then mixed with mPEG-SS-PLGA-SH solution (80 mg) containing DMF (8 mg). The whole solution was added drop-wise into 40 mL of water until PLGA-IR1048MZ-Cat nanobody (IC-NPs) was formed after 3 h stirring. IC-NPs (1 mg/mL) and 5, 5′-Dithio bis-(2-nitrobenzoic acid) (DTNB, 1 mM) were incubated together for 25 min at room temperature in order to activate the-SH group in mPEG-SS-PLGA-SH. Activated nanobody was then centrifuged and washed with PBS for 3 times. On the other hand, Nb (PEG) was processed by 5 mol α-Aminoethanethiol Hydrochloride for 1 h at 37 °C and purified by ӒKTA system (GE Healthcare) using desalination column (HiTrapTM). The purified Nb (PEG) was mixed with activated IC-NPs at a ratio of 5: 4. The redundant Nb was then removed by centrifugation after 3-h incubation at 25 °C. Final product (Nb@IC-NPs) was resuspended and washed with PBS.

#### Sizing and Morphology of Nb@IC-NPs

Size distribution of Nb@IC-NPs was measured by Zetasizer Nano ZS (Malvern Nano series, Malvern, U.K); Morphological study was performed by TEM (HT7700, HITACHI, Japan).

#### Response of Nb@IC-NPs toward NTR

UV–vis and NIR-II fluorescent spectrums were evaluated in 10 mM HEPES buffer at 37 °C. NIR-II fluorescent spectrum was acquired by scanning and emitting a spectral ranging from 1010 to 1200 nm on a FSP920 spectrofluorometer machine, with an excitation wavelength (λex) 980 nm. Fluorescent response of 5 μg/mL Nb@IC-NPs toward NTR at different concentrations (0, 1, 2, 3, 4, 5, 6, 7, 8, 9, and 10 μg/mL) (500 μM NADH as co-enzyme) was recorded respectively. All samples were examined in PBS while 1% DMSO was present as co-solvent. Each sample was repeated three times.

To investigate the selective response of Nb@IC-NPs to NTR:5 μg/mL Nb@IC-NPs and HEPES solution containing 500 μM NADH were incubated together with different active substances for 30 min at 37℃, followed by fluorescence measuring. Each sample was repeated three times. Active substance:MgCl_2_ (1 mM), NaCl (20 mM), CaCl_2_ (2 mM), KCl (20 mM), H_2_O_2_ (10 μM),-OCl (10 μM), Arginine (Arg, 2 mM), Cysteine (Cys, 2 mM), DL-Dithiothreitol (DTT, 2 mM), Glutathione (GSH, 1 mM), Glucose (50 mM), Vitamin C (2 mM), NTR (10 μg/mL).

#### Generation of O_2_

H_2_O_2_ (150 µM) was incubated together with Nb@IC-NPs (50 µg/mL IR1048-MZ) in anaerobic PBS at 37 °C, after which oxygen level was measured by an oximeter (Xylem, California, USA) in 1 h every 5 min per time.

#### Cellular uptake in vitro

NSCLC cell line A549 was cultured in DMEM medium containing 10% FBS, 100 U/mL Penicillin and 100 μg/mL Streptomycin in an incubator (5% CO_2_) at 37 °C.

A549 cells were seeded in an 8-well plate at a density of 1 × 10^4^ cells/well overnight. Then 10 μg/mL NTR was incubated with 10 µg/mL of IR1048MZ, IC-NPs and Nb@IC-NPs respectively for 10 min, after which cells were added and kept incubating for 2 h. Whole mixture was washed with PBS for twice and fixed by 4% paraformaldehyde (200 μl) for 10 min at 4 °C. Product was then washed twice again with PBS and stained with 4,6-diamidino-2-phenylindole (DAPI; 10 μg/mL in PBS) for 10 min to observe nucleus. Finally, free dye was washed off and removed by PBS and cellular uptake was observed by CLSM (Leica TCS SP8 STED, Weztlar, Germany).

Cellular uptake of Nb@IC-NPs by A549 cells was detected by flow cytometry: A549 cells were cultured in a 12-well plate at a density of 5 × 10^5^ cells/ well overnight. Next, 10 μg/mL NTR was incubated with 10 µg/mL of IR1048-MZ, IC-NPs and Nb@IC-NPs respectively for 10 min, after which cells were added for another 2 h-incubation. Redundant particles were washed off with PBS and cells were digested with trypsin. NP uptake was then quantified by flow cytometry (BD FACSCalibur, Becton, Dickinson and Company, USA).

#### Hypoxic cell culture

A549 cells were seeded in a 6-well plate at a density of 2 × 10^5^ cells/well. After 24-h culture, medium was refreshed and cells were kept in an anaerobic chamber with 94.5% N_2_, 5% CO_2_ and 0.5% O_2_ to induce a hypoxic condition. Control cells were cultured under normal condition (5% CO_2_, 95%Air).

#### Analysis of intracellular hydrogen peroxide

Intracellular H_2_O_2_ level was detected using MAK164 kit (Sigma-Aldrich). For hypoxic condition, cells were cultured in low oxygen chamber for 12 h and then incubated together with 10 µg/mL PBS, IC-NPs and Nb@IC-NPs respectively for 4 h. Medium was then replaced with kit’s testing buffer and kept incubated for another 1 h. Intracellular H_2_O_2_ was observed by CLSM after washed with PBS. For culturing under normal condition, cells were processed by 100 μM H_2_O_2_ for 12 h, and then incubated with PBS, IC-NPs and Nb@IC-NPs respectively for 4 h. The medium was replaced with testing buffer and cells were incubated for another 1 h. Intracellular H_2_O_2_ was then observed by fluorescence imaging after washed with PBS.

#### Generation of extracellular singlet oxygen

Singlet Oxygen Sensor Green (SOSG, Invitrogen, Carlsbad, CA, USA) was applied to detect singlet state oxygen under normal and hypoxic condition. A549 cells were seeded in a 6-well plate at the density of 2 × 10^5^ cells/well and cultured for 12 h. R1048MZ, IC-NPs, and Nb@IC-NPs were added into the cell at 10 µg/mL each. Cells cultured with PBS were used as control. SOSG was added 15 min before light illuminated. After 4 h incubation, NIR (980 nm, 0.1 W/cm^2^) was applied to illuminate cells for 5 min, after which, cells were washed with PBS and observed under CLSM (ex/em = 488 /525 nm).

#### Cyto-toxicity analysis

Under normal or hypoxic condition, MTT assay was performed to assess the effect of IR1048MZ, IC-NPs and Nb@IC-NPs on A549 cells with or without illumination. Cyto-toxicity was firstly tested without illumination. A549 cells were seeded on a 96-well plate at a density of 2 × 10^4^ cells/well and further cultured for 12 h. Later, IR1048MZ, IC-NPs and Nb@IC-NPs were added at different concentrations (0, 0.2, 0.5, 1, 2, 5, 10, 20 and 50 µg/mL) into cells. Three replicate wells were set at each concentration. MTT was performed to test cellular activity after another 72 h-incubation. In order to testify hypoxic cyto-toxicity under illumination, cells were seeded in a 96-well plate at a density of 2 × 10^4^ cells/well and cultured for 12 h. Cells were then transferred to anaerobic chamber and incubated for 6 h. Then IR1048MZ, IC-NPs and Nb@IC-NPs were added at different concentrations (0, 0.2, 0.5, 1, 2, 5, 10, 20 and 50 µg/mL) into cells. After 4 h culturing, NIR was applied on cells for 5 min. MTT was performed to test cellular activity after another 72-h incubation. In order to testify normal cyto-toxicity under illumination, cells were seeded in a 96-well plate at a density of 2 × 10^4^ cells/well and cultured for 18 h. IR1048MZ, IC-NPs and Nb@IC-NPs were added at different concentrations (0, 0.2, 0.5, 1, 2, 5, 10, 20 and 50 µg/mL) into cells. After 4-h culture, NIR was applied on cells for 5 min. MTT was performed to test cellular activity after another 72-h incubation. Cellular activity (%) = [OD490 (sample)-OD490 (blank)]/[OD490 (control)-OD490 (blank)].

#### Cell apoptosis

Prior to 24 h of treatments by 10 µg/mL of IR1048MZ, IC-NPs and Nb@IC-NPs, A549 cells were seeded into a 6-well plate at a density of 1 × 10^6^ cells/well overnight (Steps for laser group were as before). For flow cytometry analysis, cells were digested by trypsin after washed with PBS twice, after which all were collected in a tube and resuspended in a specific annexin-binding buffer (100 μL/per tube). Alexa Fluor 488 annexin V (5 μL) and 100 μg/mL PI solution (1 μL) were added into cell suspension (100 μL) and incubated together for 30 min at 25 °C. After incubation, annexin-binding buffer (400 μL) was added into each sample, followed by mixing on ice. Apoptosis was then immediately observed by flow cytometry (Becton, Dickinson and Company) and apoptotic cells were calculated as the sum of early apoptosis cells (ANNEXIN V^−^/PI^+^) and later apoptosis cells (ANNEXIN V^+^/PI^+^). For optic imaging, cells were directly observed by stereo microscope (Olympus, Tokyo, Japan). CLSM imaging was performed after cells were washed with PBS and stained using an apoptosis kit.

#### Live/dead cell staining

A549 cells were seeded in a 8-well plate at a density of 3 × 10^4^ cells/well and then treated with 10 µg/mL of IR1048MZ, IC-NPs and Nb@IC-NPs (Steps for laser group were as before). Next, 2 μM Calcein and 50 μg/mL PI were used for cell staining for 10 min before observation by CLSM.

#### Animal culturing and tumor model

BALB/c female mice (4–6 weeks) were purchased from SJA Laboratory Animal Co. Ltd, (Hunan, China) permitted by Institutional Animal Care and Use Committee (IACUC). To construct subcutaneous tumor model, 100 μL PBS containing A549 cells (1 × 10^6^ cells) were injected into left inguinal area of the BALB/c mice. For a lung metastasis model, the procedure was done as subcutaneous tumor model, followed by a second round of A549 cells (2 × 10^5^ cells) injection via tail vein after 6 days. Tumor size = width^2^ × length × 0.5

#### In vivo NIR-II and photoacoustic hypoxic imaging of Nb@IC-NPs

In order to assess NIR-II fluorescence imaging and bio-distribution in vivo, A549 tumor-bearing BALB/c mice were anesthetized by intraperitoneally injected with 40 mg/kg pentobarbital sodium. Nude mice were euthanized 14 h after 100 μL Nb@IC-NPs (5 mg/kg IR1048MZ) was injected through tail vein. Tumor and main organs (heart, liver, spleen, lung, and kidney) were collected for NIR-II imaging analysis. NIR-II fluorescence imaging of the whole body was obtained at given times (0, 5, 10, 14, 20, 24 and 48 h) after Nb@IC-NPs were injected. NIR-II fluorescence imaging was accomplished by a 640 × 512 pixel 2D InGaAs/SWIR VGA standard camera (Photonic Science, UK) with a long-pass filter (Thorlabs FEL1000 nm). Laser beam excitation was performed with a 980 nm laser transmitter (MDL-III-980R, Changchun New Industries Optoelectronics Technology Co., Ltd.).

PA imaging was observed by ENDRA's preclinical photoacoustic CT scanner (Endra Nexus 128). Wavelength for impulse excitation was set at 880 nm. Tumor zones and their corresponded signal intensities of PA images were analyzed at different given times (0, 5, 10, 14, 20, 24 and 48 h). Penetration depth of PA imaging was measured in the longitudinal section (L) 14 h post-injection.

#### Measurement of Singlet oxygen and assessment of in vivo hypoxia

In order to evaluate tumor oxygenation of Nb@IC-NPs, tumor-bearing mice were injected with PBS, IR1048MZ (5 mg/kg) and Nb@IC-NPs (5 mg/kg IR1048MZ) intravenously. Thereafter, 3 h after injection, 60 mg/kg Pimonizole hydrochloride (Hypoxyprobe-1plus kit, Hypoxyprobe Inc.) was delivered by intraperitoneal injection. Tumors from all groups were collected after 1 h. The collected samples were sliced and incubated firstly with immunoglobulin G (IgG)1 (mouse anti-pimonidazole) and then with IgG2 (Alex 488-conjugated goat anti-mouse, KPL, USA). After stained by DAPI, sample sections were observed by CLSM. PBS, IR1048MZ (5 mg/kg) and Nb@IC-NPs (5 mg/kg IR1048MZ) were injected into tumor-bearing mice intravenously for each group. After 13.5 h, SOSG (6.25 μg/mouse) was delivered by peritoneal injection. For laser-treating group, samples were illuminated by a 980 nm laser transmitter for 30 min (0.1 W/cm^2^). Tumor sample from each group were then collected for cryo-sectioning, after which SOSG oxidative signal was observed by CLSM.

#### HIF-1α immunostaining

Recombinant anti-HIF-1α antibody (Alexa Fluor® 488) (ab190197) (Abcam, Cambridge, UK) was used to evaluate intra-and extra-cellular hypoxic level. In vitro studies were conducted by incubating A549 cells under both normal and hypoxic conditions, followed by further incubation with PBS, IC-NPs (10 µg/mL IR1048-MZ) and Nb@IC-NPs (10 µg/mL IR1048-MZ), respectively for 4 h. Hypoxic PDT was performed in an anaerobic chamber. Then cells were stained by anti-HIF-1α (ab190197, Abcam) and DAPI, and then observed by CLSM. For in vivo immunofluorescence imaging, A549 tumor-bearing mice were treated with IR1048MZ, IC-NPs or Nb@IC-NPs, while PBS was used as a control. Tumors were excised after treatment and dried for at least 1 h before being fixed in acetone for 10 min at 20 ℃. Samples were stained by Alexa Fluor®488-HIF-1α and DAPI, washed with PBS twice, and then observed by CLSM.

#### In vitro anti-tumor activity studies

7 days after tumor was inoculated subcutaneously, PBS, IR1048MZ (5 mg/kg), IC-NPs (5 mg/kg IR1048-MZ) and Nb@IC-NPs (5 mg/kg IR1048-MZ) were delivered via tail vein into A549 tumor-bearing mice (n = 5). For laser treating group, 14 h after different components were injected, tumor was exposed to 980 nm laser for 30 min (0.1 W/cm^2^). Tumor size and weight were monitored every 3 days. For anti-metastasis activity of Nb@IC-NPs, 1 day after A549 cells were injected intravenously, PBS, IR1048-MZ (5 mg/kg) and Nb@IC-NPs (5 mg/kg IR1048-MZ) were injected intravenously into A549 tumor-bearing mice (n = 5). Then, 14 h after injection, tumor was exposed to 980 nm laser for 30 min (0.1 W/cm^2^). 18 days after tumors were treated with PDT, the lungs were collected after euthanizing the mice, and the metastatic nodules were observed before applying H&E staining.

#### In vivo bio-safety of Nb@IC-NPs

Female BALB/c mice were injected with 100 μL PBS or Nb@IC-NPs (contained 5 mg/kg IR1048-MZ, n = 3) through tail vein. Alanine Amino-Transferase (ALAT) or Blood Urea Nitrogen (BUN) activity assay kit (Sigma-Aldrich) was applied at day 1 and day 7 post-injection to assess the liver or kidney function. Mice were euthanized 7 days after injection for organ collection and sectioning, after which H&E staining was applied and observed by Olympus microscope (Olympus BX53, Tokyo, Japan).

#### Statistical analysis

All experimental data were analyzed using the SPSS 21.0 software (IBM Corp. Armonk, NY, USA). Measurement data were summarized by mean ± standard deviation from at least three independent experiments. Comparison between two groups were performed by unpaired* t* test. Comparisons among multiple groups were performed by one-way analysis of variance (ANOVA) with Tukey’s post hoc test. Comparison among groups at different time points was performed using repeated measures ANOVA with Bonferroni’s post hoc test. *p* < 0.05 indicated the difference was statistically significant.

## Results

### Successful preparation and characterization of Nb@IC-NPs

**Fig. 1:**
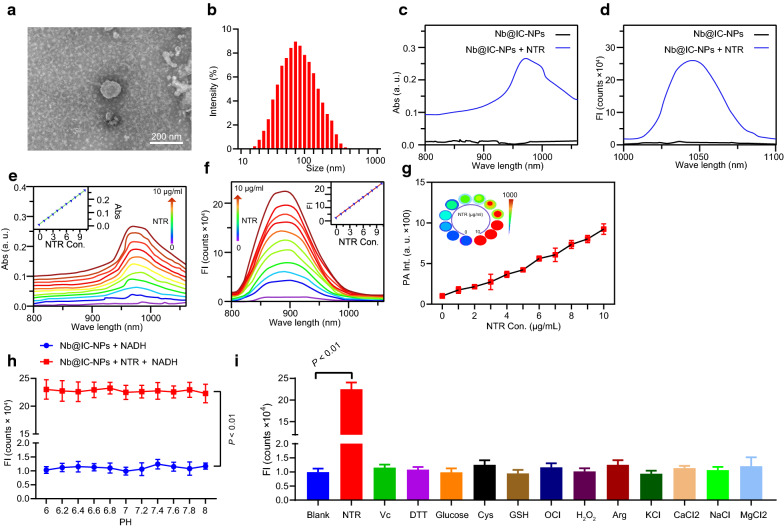
Characterization of Nb@IC-NPs. **a** TEM imaging of Nb@IC-NPs; **b** Size distribution of Nb@IC-NPs; **c** UV absorption spectrum of Nb@IC-NPs to NTR; **d** Fluorescence emission spectrum of Nb@IC-NPs to NTR; **e** UV absorption spectrum showing a concentration-dependent response of Nb@IC-NPs to NTR; **f** Fluorescence emission spectrum showing a concentration-dependent response of Nb@IC-NPs to NTR; **g** Photoacoustic signal showing a concentration-dependent response of Nb@IC-NPs to NTR; **h** Fluorescence signal change of Nb@IC-NPs in response to NTR under different PH; **i** Effect of different active substances on Nb@IC-NPs’ fluorescence signal. Comparison between two groups were performed by unpaired *t* test. Comparisons among multiple groups were performed by one-way ANOVA with Tukey’s post hoc test. Comparison among groups at different time points was performed using repeated measures ANOVA with Bonferroni’s post hoc test. *p* < 0.05 indicated the difference was statistically significant

We first synthesized IR1048-MZ. mPEG-SS-PLGA-SH was dissolved to encapsulate IR1048-MZ and Catalase (Cat) to form IC-NPs. On the other hand, Anti-EGFR-Nanobody was expressed and purified. Final Nb@IC-NPs product was formed by combining mPEG-SS-PLGA-SH and Anti-EGFR-Nanobody through desulfide bond. TEM results showed Nb@IC-NPs as homogeneous nanoparticles with a diameter of 82 ± 1.5 nm (Fig. 1a). Diameter and PDI of Nb@IC-NPs measured by Dynamic light scattering (DLS) were 85 ± 1.2 nm and 0.105, indicating a good distribution of Nb@IC-NPs (Fig. 1b). We then studied the response of Nb@IC-NPs towards NTR. Nb@IC-NPs showed an extremely weak absorbance in the spectral range of 900–1100 nm, however after being reduced by NTR, a maximum absorption peak was produced at about 980 nm (Fig. 1c). In addition, there was almost no fluorescence emission at the wavelength of 1046 nm, whereas signal was intensified after responded to NTR (Fig. 1d). It was found that the absorption peak of Nb@IC-NPs showed a linear correlation with the concentration of NTR because both absorption peak and fluorescence signals were gradually intensified when the concentration of NTR increased by increments of 1 μg/mL until it reached to maximum of 10 μg/mL (Fig. 1e, f). Moreover, NTR initiated reduction process of Nb@IC-NPs resulted in their intensification near infrared absorption, which as expressed as the stronger photoacoustic signals. (Fig. 1g). When selectivity toward NTR was further investigated, it was found that Nb@IC-NPs exhibited a stable response to NTR, as PH and other active substances showed no interference. As such, application of our design in a more complicated cellular environment was assured (Fig. 1h, i).

### Cellular uptake in vitro

**Fig. 2:**
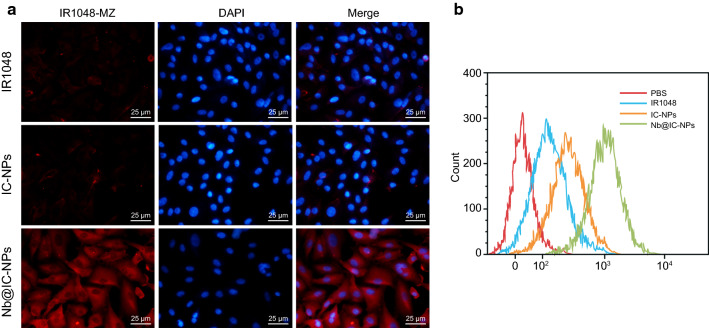
Uptake study of Nb@IC-NPs. **a** CLSM was applied to investigate the uptake efficiency of IR1048-MZ, IC-NPs and Nb@IC-NPs by A549 cells (blue indicates DAPI, red color indicates IR1048-MZ fluorescence, scale bar = 25 μm). **b** Cellular uptake efficiency of IR1048MZ, IC-NPs and Nb@IC-NPs by A549 cells confirmed by flow cytometry. n = 3

To confirm the uptake of Nb@IC-NPs by tumor cells, we initially had Nb@IC-NPs, IC-NPs and IR1048-MZ activated by NTR, and then incubated them with A549 cells to observe uptake efficiency. Results showed almost no apparent changes between IR1048-MZ and IC-NPs groups, while multiple strong fluorescence signals (shown as red) were observed in the cells treated with Nb@IC-NPs (Fig. 2a). Meanwhile, red fluorescence signal can neither be discovered in MCF-7cells (data were not shown or see Additional file 1: Figure S1, Additional file 2: Figure S2, Additional file 3: Figure S3), which suggested an uptake specificity of such nanocomplex toward A549 cells. Flow cytometry was also applied to analyze uptake efficiency of different nanoparticles by A549 cells, identifying a much higher cellular uptake preference toward Nb@IC-NPs than either IC-NPs or IR1048-MZ (Fig. 2b). To sum up, the introduction of Anti-EGFR-Nanobody enhanced uptake efficiency of Nb@IC-NPs.

### Generation of extracellular singlet oxygen

**Fig. 3:**
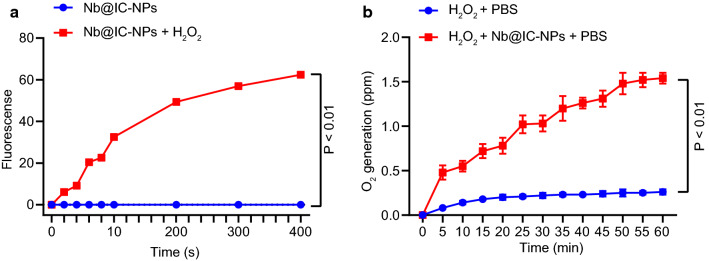
Generation of singlet oxygen induced extracellularly by Nb@IC-NPs. **a** Generation of O_2_ in Nb@IC-NPs solutions with or without H_2_O_2_ added when illuminated by 980 nm NIR; **b** Catralytic ability of Nb@IC-NPs in PBS. Comparison between two groups were performed by unpaired *t* test. Comparisons among multiple groups were performed by one-way ANOVA with Tukey’s post hoc test. *p* < 0.05 indicated the difference was statistically significant. n = 3

Since hypoxic cancer cells often come with a high concentration of H_2_O_2_, one of the most effective ways to overcome cellular hypoxia is to decompose H_2_O_2_ for the generation of more O_2_ using Catalse (CAT). For this reason, a number of studies were carried out to testify the catalytic ability of Nb@IC-NPs. First of all, in the presence of NIR, remarkable fluorescence signal was observed in Nb@IC-NPs with H_2_O_2_ added (Fig. 3a). After H_2_O_2_ was added into PBS (with or without Nb@IC-NPs at 37 °C and generation of O_2_ was detected by an oxygen sensor over the range of 1 h. The results showed a steadily increased level of O_2_ due to the catalytic ability of Nb@IC-NPs (Fig. 3b). To confirm the catalytic efficiency on a cellular level, 100 μM H_2_O_2_ was added into cells and the results were determined using Fluorometric Hydrogen Peroxide Assay Kit. As anticipated, a dramatic reduction in fluorescent intensity was observed in samples processed with Nb@IC-NPs, validating an indeed decomposition of H_2_O_2_ by our nanocomplex (Additional file 1: Fig. S1a). Results illustrated that a substantial amount of singlet oxygen as a major type of ROS can be induced by Nb@IC-NPs in either normal or hypoxic condition when exposed to 980 nm NIR (Additional file 1: Fig. S1b).

### Photodynamic toxicity

**Fig. 4:**
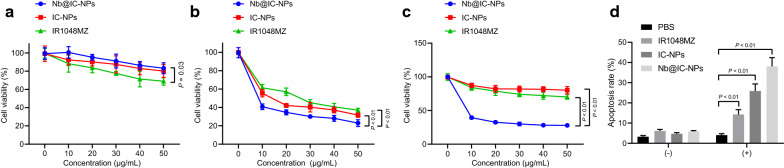
In vitro photodynamic toxicity of Nb@IC-NPs. **a** Toxicity on A549 cells caused by Nb@IC-NPs in dark; **b** Cyto-toxicity test under normal and hypoxic **c** conditions; **d** Flow cytometry analyzing the effect of different nanoparticles on A549 cell apoptosis. Comparison among groups at different time points was performed using repeated measures ANOVA with Bonferroni’s post hoc test. *p* < 0.05 indicated the difference was statistically significant. n = 3

In subsequent work, we planned to evaluate the photodynamic toxicity of Nb@IC-NPs to A549 cells when exposed to 980 nm laser. Cellular activity was measured by MTT assay. Results revealed a moderate level of cytotoxicity in the absence of the laser treatment after applying Nb@IC-NPs, which might be due to the generation of singlet oxygen during the Cat catalyzation. (Fig. 4a). Additionally, concentration-dependent toxicity was discovered with the presence of laser in all three groups, but Nb@IC-NPs-based PDT exerted the most lethal effect on cancer cells. (Fig. 4b, c). Similar results were observed using flow cytometry and the cell apoptosis was calculated as the sum of a percentage of early apoptotic cells (Q4) and late apoptotic cells (Q2). Apoptosis was barely occurred in the groups without laser illumination, while high level of cellular apoptosis was induced by nanocomplex in laser-treated groups. However, Nb@IC-NPs groups suggested a more aggressive apoptosis rate than any other groups (Fig. 4d). This was also confirmed by microscopic images, where substantial dead cells can be found in only NIR-treated Nb@IC-NPs groups (Additional file 1: Fig. S1c). Next, CLSM was used to assess the cyto-toxicity of Nb@IC-NPs under 980 nm laser illumination and cells were stained by Apoptosis Detection Kit. It was discovered that cells in non-illuminated groups possessed a good uniformity regarding size distribution and morphology, however after being treated by NIR, apoptosis was observed in all three groups (IR1048-MZ, IC-NPs and Nb@IC-NPs), where apoptosis level was lower in IC-NPs group than that in IR1048-MZ group. An explanation for this outcome might be the existence of Cat that led to an increased toxicity due to the generation of ROS. The most severe and abundant apoptosis situations were present in the group with formulated Anti-EGFR-Nanobody and laser application (Additional file 1: Fig. S1d). All results illustrated that the photodynamic effect of Nb@IC-NPs can cause A549 cell death.

### In vivo NIR-II and hypoxia imaging

**Fig. 5:**
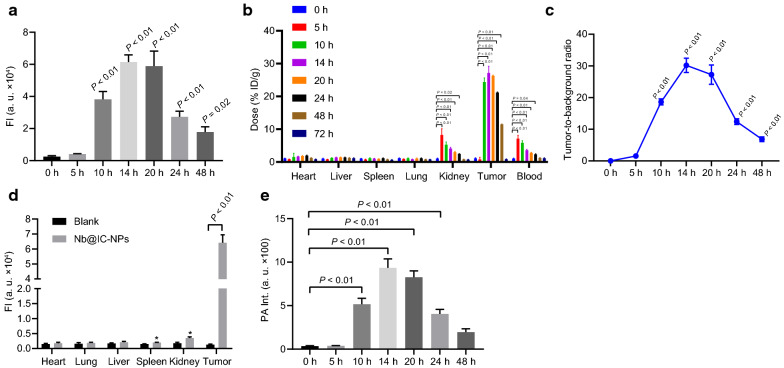
In vivo NIR-II/PA imaging of Nb@IC-NPs. **a** Fluorescence intensity analysis; **b** In vivo bio-distribution of Nb@IC-NPs; **c** Quantitative analysis of fluorescence intensity; **d** Quantitative analysis of PA intensity; **e** Penetration depth of PA signals after Nb@IC-NPs was injected; Comparisons among multiple groups were performed by one-way ANOVA with Tukey’s post hoc test. *p* < 0.05 indicated the difference was statistically significant. n = 3

Owing to the auto-fluorescence and decreased photon scattering phenomenon in biological system, NIR-II fluorescence imaging possesses the advantage of reaching to the maximum depth of penetration in tissue. In this article, the feasibility of achieving NIR-II imaging by using Nb@IC-NPs was verified by injecting such nanocomplex into A549 tumor-bearing (NTR was expressed in hypoxia) BALB/c nude mice through tail vein. After injection, NIR-II fluorescence signal could be clearly observed in tumor tissue, and intensity peak was reached after 14 h (Fig. 5a). Note that, Nb@IC-NPs was quite different from other probes reported by most other studies, when Nb@IC-NPs was used for NIR-II fluorescence imaging upon hypoxic tumors, background signal was not detected. Our results indicated that, among all organs treated with Nb@IC-NPs at various time points, the highest maximum accumulation of the probe occurred in the kidney at 5 h after injection, which suggested that the probe underwent renal metabolism. Aside from tumor and kidney, a high presence of probes was also found in blood, indicating the probes were absorbed into the bloodstream and transported through kidney (Fig. 5b, c). Strong NIR-II fluorescence intensity of Nb@IC-NPs was detected in tumors, whereas other organs (heart, lung, liver, spleen and kidney) showed no detectable signals (Fig. 5d, e). The specificity of Nb@IC-NPs by promoting oxygenation and preventing the autofluorescence makes Nb@IC-NPs an outstanding candidate for tumor NIR-II fluorescence imaging, even with higher potential than nanoparticles that was reported for NIR I imaging.

### Immunofluorescence staining

HIF-1α protein was generally upregulated under hypoxia resulting in accelerated tumor growth. Hence, the ability of Nb@IC-NPs to improve hypoxia was assessed by detecting the expression level of HIF-1α protein in both A549 cells and tumors, since incubating cells under hypoxia will also lead to an accumulation of HIF-1α. Compared with groups processed with PBS and IR1048-MZ under hypoxia, cells incubated with Nb@IC-NPs experienced an obvious decrease in terms of HIF-1α fluorescence signal, that quite identical to PBS-processed group under normal conditions, indicating a status where was undoubtedly improved (Additional file 2: Fig. S2a). Immunohistochemistry staining of HIF-1α further affirmed the effects of Nb@IC-NPs on the mitigation of tumor hypoxia, whereas the same result was not noticed in IC-NPs group that was lack of a specific targeting (Additional file 2: Fig. S2b). These data illustrated that Nb@IC-NPs could effectively overcome hypoxia on both cellular and tumor level, which is critical in improving tumor hypoxia as well as promoting PDT.

Next, immunofluorescence was performed using a hypoxyprobe kit to test the efficiency of generating O_2_ by Nb@IC-NPs at lesion sites so that tumor hypoxic level could be determined. In PBS and pure IR1048-MZ groups, widely distributed greed fluorescence signal indicated the general existence of hypoxic areas in tumor section. In contrast, tumor hypoxia was unnoticeable in mice injected with Nb@IC-NPs, which indicated the effect of Nb@IC-NPs on specific targeting and oxygenation (Additional file 2: Fig. S2c). Fluorescent probe (SOSG) was applied 14 h after Nb@IC-NPs injection to investigate the formation of singlet oxygen during in vivo PDT. Results showed that due to the limited accumulation of photosensitizer and lack of catalytic reaction that generated O_2_, the efficacy of PDT was not maximized in groups processed with IR1048-MZ. However, it was an opposite case in groups with Nb@IC-NPs added, where strong green fluorescence was detected, indicating sufficient ^1^O_2_ generated (Additional file 2: Fig. S2d). The target specificity of Anti-EGFR-Nanobody toward A549 cells and possible EPR effect of Nb@IC-NPs could play a critical role in the enrichment of O_2_ level and IR-1048-MZ contrast agent at the tumor site after the administration of Nb@IC-NPs. Therefore, an enhanced PDT could be achieved by sufficiently generating O_2_ in tumor hypoxia via circulating Nb@IC-NPs.

### Anti-tumor effect

**Fig. 6:**
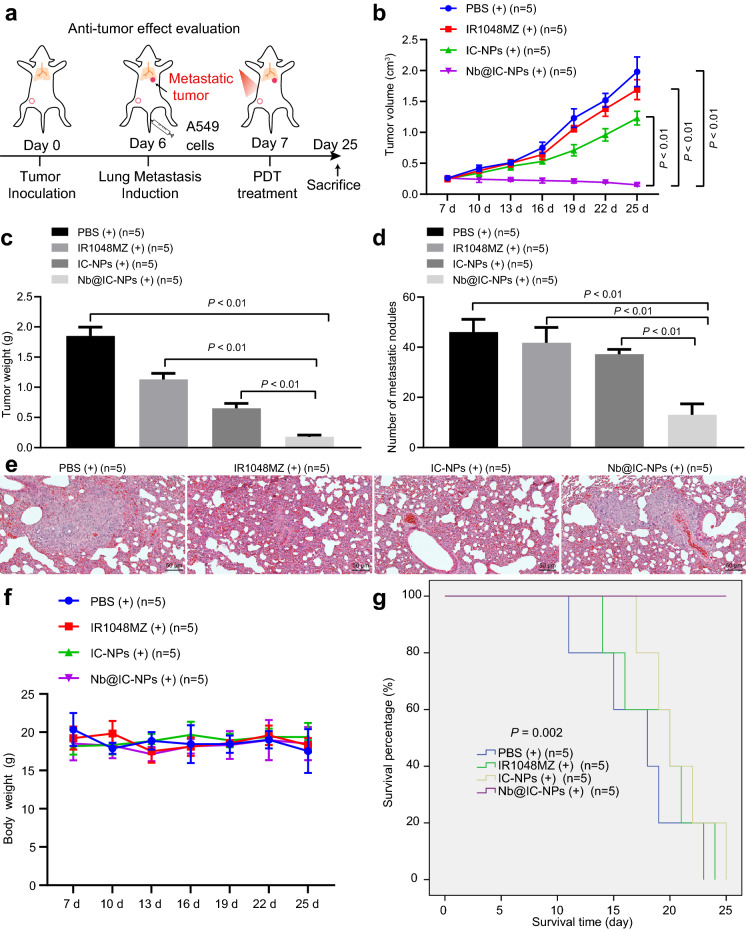
In vivo anti-tumor effect. **a** Result showing Nb@IC-NPs-based PDT inhibiting primary A549 tumor and its’ metastasis; **b** Curve of tumor growth; **c** Tumor weights on the 25th day; **d** Number of pulmonary metastatic nodules; **e** H&E staining for lungs; **f** Mice weight change; **g** Mice survival curve; Comparisons among multiple groups were performed by one-way ANOVA with Tukey’s post hoc test. Comparison among groups at different time points was performed using repeated measures ANOVA with Bonferroni’s post hoc test. *p* < 0.05 indicated the difference was statistically significant. n = 5

In the following studies, we investigated the effect of Nb@IC-NPs mediated PDT on A549 tumor by building unilateral subcutaneous tumor models and tumor metastasis models on mice. Primary tumor was grown on the left side of inguinal region by injecting A549 cells, and metastatic tumor model was initiated after 6 days by inoculating A549 cell to the tail vein (Fig. 6a). On the 7th day, primary tumor was then processed by PBS + Laser, IR1048-MZ + Laser, IC-NPs + Laser and Nb@IC-NPs + Laser. Daily tumor volume was recorded from day 7 and mice were euthanized on day 25 to evaluate metastasis of the lung. In comparison to the groups of PBS + Laser, IR1048-MZ + Laser, and IC-NPs + Laser, we found that the tumor size shrunk significantly in the combined applications of Nb@IC-NPs and laser treatment (Fig. 6b). Specifically, IC-NPs + Laser showed a better result than IR1048-MZ + Laser, however worse than Nb@IC-NPs + Laser, regarding the inhibition of tumor growth, which is caused by the deficiency of effective accumulation of IC-NPs at tumor sites. Mice were euthanized at the 25th day for primary tumor collection. Th five tumor samples treated with Nb@IC-NPs + Laser weighed below 0.3 g, which were significantly under the weight levels of the other groups. Generally, tumor weights in Nb@IC-NPs + Laser group were much lower than other controls (Fig. 6c). Our results also demonstrated that the average number of pulmonary metastatic nodules in Nb@IC-NPs + Laser group (13 ± 4) was significantly lower than groups such as PBS + Laser (46 ± 5), IR1048-MZ + Laser (42 ± 6), and IC-NPs + Laser (37 ± 2) (Fig. 6d). As a result, Nb@IC-NPs-based PDT exhibited 72.3% inhibition of lung metastasis, which is much higher than the IR1048-MZ + Laser and IC-NPs + Lase groups. Results in Fig. 6e also provided direct evidence proving the anti-metastasis effect of Nb@IC-NPs + Laser group. In the meantime, H&E staining further confirmed the above results in terms of nodule amount and volume in Nb@IC-NPs + Laser group (Fig. 6f). In summary, all results we obtained point to the same conclusion that Nb@IC-NPs-based PDT could not only inhibit tumor proliferation, but also lung metastasis. Since the weight of all mice didn’t appear to be significantly different, it is believed that the Nb@IC-NPs-based PDT is tolerable for mice. Most importantly, compared with all controls, the survival rate of mice treated with Nb@IC-NPs illuminated by NIR reached as high as 100% (Fig. 6g).

### Toxicological analysis

Bio-safety is essential to an excellent method for tumor treatment. Here we injected Nb@IC-NPs into mice through tail vein, followed by organ collection and blood analysis for physiological toxicity. H&E tissue staining results demonstrated that Nb@IC-NPs showed no sign of the damage in the major organs of the mice (Additional file 3: Fig. S3). Results of blood routine test showed that, compared with healthy groups, multiple blood indicators were in normal range 1 and 7 days after treated with Nb@IC-NPs (Table 1). More importantly, indicators such as Alanine Transaminase (ALT), Alkaline Phosphatase (ALP) and Aspartate Aminotransferase (AST) showed no obvious change after treated with Nb@IC-NPs, displaying zero side effect to liver and kidney functions (Table 1). These results reveal that Nb@IC-NPs is a safe nano-platform for tumor treatment that can be normally and effectively metabolized by the body. Meanwhile, the possibility of treating other tumors using our nanocomplex could be further explored, whereas a systematic perfection should be accomplished before being applied clinically.

**Table 1:** ALB/c mice injected with Nb@IC-NPs was sacrificed on the 1st and 7th day

	WBC(10^9^/L)	RBC(10^12^/L)	HGB(g/L)	HCT(%)	MCV(fL)	MCH(pg)
Reference range	5.64–14.85	8.16–11.69	124 –189	43 –67	50.8 –64.1	13 – 17.6
Healthy control	5.25 ± 1.12	10.36 ± 0.41	154.0 ± 5.6	49.0 ± 2.4	47.3 ± 0.6	14.7 ± 0.3
1D after treatment	6.08 ± 1.10	8.90 ± 2.57	158.2 ± 35.6	41.5 ± 12.9	46.1 ± 1.3	14.4 ± 0.6
7D after treatment	6.50 ± 1.65	10.8 ± 0.61	159.1 ± 8.5	50.2 ± 2.1	46.6 ± 0.8	14.6 ± 0.4

	MCHC(g/L)	PLT(10^9^/L)	ALT(U/L)	ALP(U/L)	AST(U/L)
Reference range	239 – 331	476 – 1611	40 – 170	108 – 367	67 –381
Healthy control	312.2 ± 2.5	914.7 ± 226	52.4 ± 10.5	159.4 ± 49.2	226.0 ± 14.4
1D after treatment	309.3 ± 9.8	821.4 ± 309	52.2 ± 14.2	163.5 ± 41.0	180.9 ± 15.2
7D after treatment	316.1 ± 4.2	1168 ± 257	65.6 ± 10.1	180.4 ± 47.4	177.5 ± 24.1

Healthy Mice without processing was used as control. Complete blood count: *WBC* White Blood Cells, *RBC* Red Blood Cells, *HGB* Hemoglobin, *HCT* Hematocrit, *MCV* Mean Corpuscular Volume, *MCH* Mean Corpuscular Hemoglobin, *MCHC* Mean Corpuscular Hemoglobin Concentration. Serum Biochemical Parameters: *ALT* lanine Transaminase, *ALP* Alkaline Phosphatase, *AST* Aspartate Aminotransferase

## Discussion

Hypoxia is a hostile characteristic of solid tumors, [42] and be recognized as one of the vital impairs of PDT effectiveness [23], leading a low therapeutic efficiency of cancer therapy [25]. During the current study, NTR enzyme-responsive nanoparticle Nb@IC-NPs was constructed by combination of anti-EGFR nanobody with photosensitizer IR1048MZ and Cat, which were used for NIR-II/PA imaging and PDT of lung cancer. Collectively, the experiments verified that Nb@IC-NPs with a favorable bio-safety could induce the efficient oxygen supply of A549 tumor, decrease the HIF-1α expression, and regulate the hypoxia microenvironment, thereby enhancing the fluorescence brightness of NIR-II/PA imaging and PDT efficacy of lung cancer.

Nano-drug delivery system is mainly accumulated in the tumor through the EPR effect [43], which influenced by properties of nanoparticles including particle size, surface charge, and shape, and among which particle size is a main factor [44, 45]. Nanoparticles with sizes of ≈ 100–150 nm possess great advantage in improving pharmacokinetics and prolonging the blood circulation [46]. Nb@IR-NPs with excellent distribution promoted the EPR effect-mediated accumulation and size-mediated penetration. Subsequently, IR1048-MZ in Nb@IC-NPs was sensitive to NTR [39]. In our study, the fluorescence intensity of deducted Nb@IC-NPs was increased linearly with the concentration of NTR. The NTR level is reported to directly relate to the degree of hypoxia in solid tumor [47]. Thus, the Nb@IR-NPs are promise agents used to evaluate the hypoxic degree of tumor. Nb@IC-NPs exhibited concentration-dependent photodynamic toxicity to A549 lung cancer cells under 980 nm laser. Considering the high concentration of H_2_O_2_ in solid tumor [48] and the ability of Nb@IC-NPs to catalyze the decomposition of H_2_O_2_ to form O_2_, we believe the photodynamic toxicity of Nb@IC-NPs was induced by Cat enzymatic formation of singlet oxygen.

Because of diminished tissue autofluorescence and reduced photon scattering without high level of light absorption, NIR-II fluorescence imaging in vivo allows deep tissue penetration and high-clarity fluorescence imaging into a living body [49, 50]. Notably, NIR-II fluorescence imaging of hypoxic tumors using Nb@IC-NPs in current investigation showed no observable background signals in vivo, promoting Nb@IC-NPs to be a promising tumor NIR-II fluorescence imaging nanoparticles. PA imaging, which relies on ultrasound signals generated by photothermal expansion of light-absorbing tissues or contrast probes under pulsed laser irradiation, has already moved into clinical trials owing to its significantly improved in vivo imaging depth (as deep as ∼12 cm) and spatial resolution compared with traditional optical imaging modalities [48]. NTR-activated PA imaging under hypoxia achieves deep tissue penetration with high spatial resolution. Therefore, Nb@IC-NPs can be used as a specific hypoxia-activated NIR-II/PA imaging nanoplatform for tumor precision detection.

HIF-1α, as a key transcription factor, is perceived to be an attractive regulator of major adaptive responses to hypoxia in tumors and involved in many pathways related to angiogenesis and metastasis [51, 52]. After treatment with Nb@IC-NPs, the HIF-1α fluorescence signal was reduced significantly, demonstrating Nb@IC-NPs could effectively overcome hypoxic characteristic of cell-to-tumor, and this is the key to improving tumor hypoxia microenvironment and promoting PDT. Targeting of anti-EGFR Nb in Nb@IC-NPs to lung cancer promote the accumulation of IR1048MZ [34, 35], resulting in precise and sufficient tumor oxygen production to retard tumor hypoxia and enhance PDT of lung cancer.

Additionally, cancer metastasis refers to the migration of cancer cells from a primary tumor to seed secondary tumors in distant sites and causes high mortality, and is difficult to treat because of the poor delivery efficiency of drugs [53]. Current tumor therapy investigation showed that 33% of A549 tumors were completely eliminated after the treatments of Nb@IC-NPs based PDT, and the remaining mice tumor of Nb@IC-NPs + Laser group were also much lighter than that of other control groups. What’s more, metastasis nodules in lung of Nb@IC-NPs based PDT were significantly less than other treatment groups. These results evidenced that Nb@IC-NPs based PDT can not only eliminate primary tumors, but also inhibit tumor metastasis, extending the lifetime of mice with lung cancer. In addition, there was no significant difference in body weight and hepatorenal function indicators of mice in these experimental groups, indicating the biosecurity of Nb@IC-NPs.

**Fig. 7:**
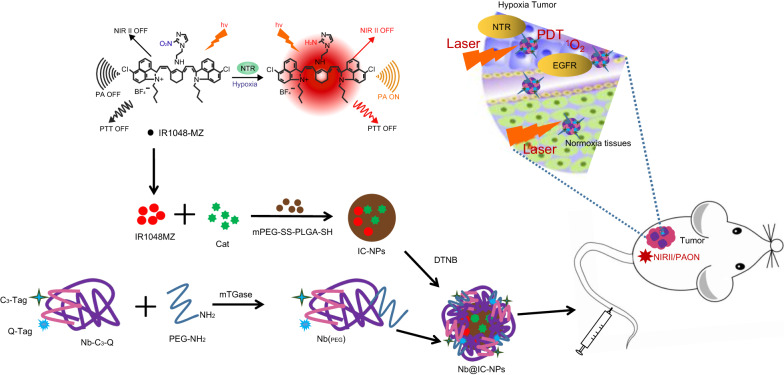
Schematic illustration of how Nb@IC-NPs improving A549 tumor hypoxia during PDT

To conclude, NIR-II/PA imaging-guided PDT nanoparticles Nb@IC-NPs were constructed by combining anti-EGFR Nb with photosensitizer IR1048MZ and Cat for therapy of lung cancer (Fig. 7). Nb@IC-NPs mediated enzymatic oxygen supply to A549 tumors promotes the decrease of HIF-1α expression. In addition, Nb@IC-NPs responded to NTR in tumor tissue, resulting in enhanced NIR-II fluorescence signal, which enables tumor diagnosis and hypoxia imaging of the tumor at the same time. Given the efficient inhibition of subcutaneous A549 tumor growth and prevention of lung metastasis, this designed strategy of Nb@IC-NPs based PDT offers a new guideline for treatment of NSCLC.

## Conclusion

In this study, we successfully constructed a nanocomplex (Nb@IC-NPs) formulating nanobody, photosensitizer (IR1048-MZ) and Catalase (Cat) for the application of PDT under the guidance of NIR-II/PA imaging. Nb@IC-NPs possess favorable bio-safety. It can improve tumor hypoxia through catalytic reaction and lowers the expression level of HIF-1α. Nb@IC-NPs can also lead to an intensified NIR-II fluorescence signal by responding to NTR at tumor sites so that tumor diagnosis and hypoxia imaging could be achieved. Nb@IC-NPs-based PDT could efficiently inhibit A549 tumor proliferation, prevent lung metastasis, and prolong mice survival cycle. In conclusion, our study illustrated that Nb@IC-NPs not only have the therapeutic potentials to effectively enhance PDT and multifunctional imaging, but also provide new ideas for treating other tumors.

## Supplementary information

**Additional file 1: Figure S1.** Generation of singlet oxygen induced extracellularly by Nb@IC-NPs and In vitro photodynamic toxicity of Nb@IC-NPs. **a** Using SOSG for the detection of the level change of H_2_O_2_ in solution with both Nb@IC-NPs and A549 incubated under normal and hypoxic conditions; **b** Using SOSG for the detection of O_2_ in PBS, IR1048-MZ, IC-NPs or Nb@IC-NPs solutions that had A549 incubated; **c** Microscopic images showing A549 cells processed with different nanoparticles; **d** Apoptosis staining images of A549 cells.

**Additional file 2: Figure S2.** Nb@IC-NPs overcoming cellular and tumor hypoxia. **a** CLSM imaging of nucleus green fluorescence showing the expression level of HIF-1α, scale bar = 25 μm; **b** Evaluation of in vivo expression level of HIF-1α using tumor section, scale bar = 50 μm; **c** Immunofluorescence imaging by hypoxia staining using tumor section of all groups, green indicates hypoxic areas; **d** Assessment of in vivo level of singlet oxygen using SOSG probe (green) after cells processed with PBS, IR1048-MZ and Nb@IC-NPs respectively, followed with an exposure to 980 nm laser illumination (30 min, 0.1 W/cm^2^). n = 3.

**Additional file 3: Figure S3.** 7 days after BALB/c mice was injected with PBS or Nb@IC-NPs (n = 3), H&E staining was performed to evaluate bio-safety upon main organs (Heart, liver, spleen, lung, kidney).

## Data Availability

The data used to support the findings of this study are available from the corresponding author upon request.

## Abbreviations

PDT: Photodynamic therapy
ROS: Reactive oxygen species
NTR: Nitroreductase
NSCLC: Non-small cell lung cancer
NP: Nanoparticle
PDT: Photodynamic therapy
EPR: Enhanced permeability and retention
EGFR: Epidermal growth factor receptor
Nbs: Nanobodies
PA: Photoacoustic
